# Supplementary material for: Identification of early fruit development reference genes in plum
Source: PLoS One. 2020 Apr 17;15(4):e0230920. doi: 10.1371/journal.pone.0230920 (PMC7164607; doi:10.1371/journal.pone.0230920)
Supplement: S1 Table — (DOCX) [file pone.0230920.s002.docx]

| Table S1. Plum RNA libraries used for RNAseq. | | | | |
| --- | --- | --- | --- | --- |
| **Sample #** | **Cultivar** | **Tissue** | **DAB** | **Reads Mapped to Peach Genome** |
| 1 | Cacanska Lepotica | Ovary | -3 | 2,368,184 |
| 2 | Cacanska Lepotica | Ovary | 0 | 1,960,178 |
| 3 | Cacanska Lepotica | Fruit | 5 | 2,568,868 |
| 4 | Cacanska Lepotica | Fruit | 8 | 9,219,065 |
| 5 | Cacanska Lepotica | Endocarp | 28 | 22,657,027 |
|  |  |  |  |  |
| 6 | ReineClaude de Bavay | Ovary | -10 | 28,265,432 |
| 7 | ReineClaude de Bavay | Ovary | 0 | 4,901,638 |
| 8 | ReineClaude de Bavay | Fruit | 3 | 5,300,822 |
| 9 | ReineClaude de Bavay | Fruit | 7 | 6,157,617 |
| 10 | ReineClaude de Bavay | Endocarp | 35 | 47,839,625 |
|  |  |  |  |  |
| 11 | Stoneless1 | Ovary | -3 | 15,442,385 |
| 12 | Stoneless1 | Ovary | 0 | 9,898,488 |
| 13 | Stoneless1 | Fruit | 4 | 21,363,580 |
| 14 | Stoneless1 | Fruit | 8 | 19,872,646 |
| 15 | Stoneless1 | Endocarp | 35 | 16,771,963 |
|  |  |  |  |  |
| 16 | Stoneless2 | Ovary | -3 | 26,827,175 |
| 17 | Stoneless2 | Ovary | 0 | 12,902,615 |
| 18 | Stoneless2 | Fruit | 4 | 12,494,037 |
| 19 | Stoneless2 | Fruit | 8 | 12,335,735 |
| 20 | Stoneless2 | Endocarp | 35 | 30,518,238 |
