# Supplementary material for: Identification of early fruit development reference genes in plum
Source: PLoS One. 2020 Apr 17;15(4):e0230920. doi: 10.1371/journal.pone.0230920 (PMC7164607; doi:10.1371/journal.pone.0230920)
Supplement: S2 Table — (DOCX) [file pone.0230920.s003.docx]

| Table S2. Expression values of Prunus genes in RNAseq libraries. | | | | | | | | | |
| --- | --- | --- | --- | --- | --- | --- | --- | --- | --- |
| Source | Peach Transcript | Function | Best Arabidopsis Match | Avg RPM | StDev RPM | Highest RPM | Lowest RPM | High/Low RPM | CV (SD/ mean) |
| Kim et al | ppa008496 | unknown protein | AT2G27285.1 | 31 | 14 | 64 | 9 | 7.30 | 0.45 |
|  | ppa009354 | peptidyl-prolyl cis-trans isomerase | AT5G35100.1 | 28 | 10 | 53 | 11 | 5.01 | 0.35 |
|  | ppa005702 | elongation factor 1-alpha / EF-1-alpha | AT5G60390.3 | 4,527 | 1,362 | 7,242 | 2,525 | 2.87 | 0.30 |
|  | ppa006366 | RAP2.12; DNA binding / transcription factor | AT1G53910.2 | 792 | 341 | 1,692 | 400 | 4.23 | 0.43 |
|  | ppa010308 | AGL8 (agamous-like 8) | AT5G60910.1 | 127 | 43 | 232 | 51 | 4.56 | 0.34 |
|  | ppa003026 | SAND family protein | AT2G28390.1 | 62 | 16 | 94 | 39 | 2.42 | 0.26 |
|  | ppa002490 | Phosphoenolpyruvate carboxykinase |  | - | - | - | - | - | - |
|  | ppa009114 | PP2A-2; protein serine/threonine phosphatase | AT1G10430.1 | 113 | 18 | 159 | 90 | 1.77 | 0.16 |
|  | ppa008371 | RAN3 (RAN GTPASE 3) | AT5G55190.1 | 549 | 120 | 951 | 417 | 2.28 | 0.22 |
|  | ppa005184 | RCA ribulose-1,5-bisphosphate carboxylase | AT2G39730.1 | 8 | 5 | 21 | 2 | 8.79 | 0.56 |
|  | ppa007294 | SAMDC (S-ADENOSYLMETHIONINE DECARBOXYLASE) | AT3G02470.4 | 43 | 30 | 147 | 17 | 8.48 | 0.69 |
|  | ppa009483 | TIP41-like family protein | AT4G34270.1 | 38 | 4 | 45 | 31 | 1.46 | 0.11 |
|  | ppa004884 | TUB8; structural constituent of cytoskeleton | AT5G23860.1 | 1,782 | 581 | 2,636 | 961 | 2.74 | 0.33 |
|  | ppa012730 | PEX4 (PEROXIN4)ubiquitin-protein ligase | AT5G25760.2 | 39 | 12 | 69 | 26 | 2.67 | 0.31 |
|  | ppa005503 | UBQ10 (POLYUBIQUITIN 10); protein binding | AT4G05320.4 | 2,908 | 667 | 4,186 | 1,342 | 3.12 | 0.23 |
|  | ppa009826 | unknown protein | AT1G31300.2 | 24 | 14 | 60 | 12 | 5.05 | 0.59 |
|  | ppa012654 | ELF5A-1 (EUKARYOTIC ELONGATION FACTOR 5A-1) | AT1G13950.1 | 432 | 81 | 541 | 251 | 2.15 | 0.19 |
| Kou et al | ppa013501 | MUB6 (MEMBRANE-ANCHORED UBIQUITIN-FOLD PROTEIN 6) | AT1G22050.1 | 14 | 8 | 44 | 5 | 8.02 | 0.57 |
|  | ppa006070 | unknown protein | AT4G14950.1 | 19 | 3 | 24 | 14 | 1.75 | 0.14 |
|  | ppa008104 | ATPK3; serine/threonine kinase | AT5G08160.1 | 105 | 39 | 209 | 59 | 3.52 | 0.37 |
|  | ppa006192 | RPT5A (REGULATORY PARTICLE TRIPLE-A ATPASE 5A) | AT3G05530.1 | 143 | 18 | 189 | 119 | 1.59 | 0.13 |
|  | ppa002870 | iqd33 (IQ-domain 33); calmodulin binding | AT5G35670.1 | 351 | 46 | 431 | 277 | 1.55 | 0.13 |
|  | ppa014541 | 40S ribosomal protein S28 (RPS28B) | AT5G03850.1 | 334 | 140 | 613 | 124 | 4.94 | 0.42 |
|  |  |  |  |  |  |  |  |  |  |
| You et al | ppa006083 | HAP13 (HAPLESS 13); protein binding | AT1G60780.1 | 161 | 40 | 250 | 113 | 2.21 | 0.25 |
|  | ppa005912 | clathrin adaptor complexes medium subunit family protein | AT5G46630.1 | 105 | 29 | 179 | 65 | 2.76 | 0.28 |
