# Supplementary material for: Identification of early fruit development reference genes in plum
Source: PLoS One. 2020 Apr 17;15(4):e0230920. doi: 10.1371/journal.pone.0230920 (PMC7164607; doi:10.1371/journal.pone.0230920)
Supplement: S3 Table — (DOCX) [file pone.0230920.s004.docx]

| Table S3. Developmental series of plum fruit and endocarp tissues. | | |
| --- | --- | --- |
| Tissue Name | Tissue Description-all from ‘Reine Claude de Bavay’ with exceptions noted | Date Collected |
| DS1 | Whole floral bud without bud scales | 4/8/2013 |
| DS2 | Carpels only from white-tip floral buds | 4/10/2013 |
| DS3 | Whole fruit 14 DAB | 4/25/2013 |
| DS4 | Whole fruit 27 DAB | 5/8/2013 |
| DS5 | Whole fruit 32 DAB | 5/13/2013 |
| DS6 | Whole fruit 40 DAB | 5/21/2013 |
| DS7 | Whole fruit 47 DAB (stone just beginning to lignify) | 5/28/2013 |
| Stendo1 | Endocarp (stone) from ‘Stoneless’ | 5/4/2010 |
| Stendo2 | Endocarp (stone) from ‘Stoneless’ | 5/10/2010 |
| Cendo1 | Endocarp (stone) | 5/4/2010 |
| Cendo2 | Endocarp (stone) | 5/10/2010 |
