# Supplementary material for: Identification of early fruit development reference genes in plum
Source: PLoS One. 2020 Apr 17;15(4):e0230920. doi: 10.1371/journal.pone.0230920 (PMC7164607; doi:10.1371/journal.pone.0230920)
Supplement: S4 Table — (DOCX) [file pone.0230920.s005.docx]

| Table S4. Primer pairs for reference genes and endocarp varying genes. | | | | | |
| --- | --- | --- | --- | --- | --- |
| Name | Peach homolog | Gene/Function | Forward Primer | Reverse Primer |  |
| P1 | ppa004809 | CorA-like/ MRS2-3-magnesium transporter | CACCAAACCCGCCAAAAC | CTCTGCCTCTTCCATGTCATAC |  |
| P2 | ppa009591 | IGPD/imidazoleglycerol-phosphate dehydratase | GTATGACACTCCACATCCGG | CACCTTTTGAACTTGGCACAG |  |
| P3 | ppa005747 | HAM1/Histone AcetylTransferase (MYST Family 1) | TTTGGCATGTATCCTCACCC | GCTTAGCAATCCTAGGTCAGAG |  |
| P4 | ppa017220 | Ras-related GTP-binding | GCTTACTATCGTGGAGCCATG | ATGTCAGCCTTGTTCCCTAC |  |
| P5 | ppa006628 | pfkB-type/ carbohydrate kinase family | TCGAAAGATGACAAGTCCCTG | AACAGCCACATTAGCAGGAG |  |
| P6 | ppa004662 | /tetratricopeptide repeat containing protein | CAGCAGCAAAGAACAAAGGAATGG | TGACAAGACCTCTGCAACAG |  |
| P7 | ppa002552 | SNX1 /SORTING NEXIN 1; phosphoinositide binding | TTTTGGTTTTGATGTGGCGG | CCTCCCACTTTTCCTTCTCTTTC |  |
| P8 | ppa006076 | PECT1/Phosporylethanolamine Cytidylyltaranserase 1 | CCTGATGGAACTGACGCTTATG | ACAAAGAAGCATACGACCAAC |  |
| P9 | ppa002787 | SDH1-1/succinate dehydrogenase | GCAGCCATAGGACTTTCAGAG | ATGTGCCATCTCCAGTCATC |  |
| *UBQ* | ppa007117 | UBQ10/Polyubiquitin 10 | AAGGCTAAGATCCAAGACAAAGAG | CCACGAAGACGAAGCACTAAG |  |
| PT1 | ppa002343 | HDG11/HOMEODOMAIN GLABROUS 11 | CGACAAGACACAAACCCATCG | CTCCAAACTCACAACCAGGTGC |  |
| PT2 | ppa020589 | FER (FERONIA)/ protein kinase | CCGAATCCAAAGAAGCACCTC | GGAAACTGGGTTGAAATACAAGCG |  |
| PT3 | ppa023578 | ARA12/serine-type endopeptidase | TGGCTGGAAAGGTTGTCTTATGTG | GGAATGTTGAACTCTTCTGGGACC |  |
| PT4 | ppa005059 | CYP707A1/ (+)-abscisic acid 8'-hydroxylase | CAGCAAGCAAAGAGAGGATGTTG | TGGATTCAATGTCAGGGACTATGC |  |
