# Supplementary material for: Identification of early fruit development reference genes in plum
Source: PLoS One. 2020 Apr 17;15(4):e0230920. doi: 10.1371/journal.pone.0230920 (PMC7164607; doi:10.1371/journal.pone.0230920)
Supplement: S5 Table — (DOCX) [file pone.0230920.s006.docx]

| Table S5. Reference candidate gene descriptions. | | | | | | |
| --- | --- | --- | --- | --- | --- | --- |
| ID | Transcript | Avg RPM^a^ | Efficiency qPCR^b^ | R value ^c^ | RefFinder Comprehensive Rank^d^ | |
|  | UBQ10 | 352 | 91 | 0.995 | 10 | |
| P1 | ppa004809 | 64 | 113 | 0.9744 | 4 | |
| P2 | ppa009591 | 36 | 112 | 0.9976 | 3 | |
| P3 | ppa005747 | 89 | 103 | 0.9999 | 2 | |
| P4 | ppa017220 | 121 | 113 | 0.9988 | 5 | |
| P5 | ppa006628 | 96 | 122 | 0.8337 | 6 | |
| P6 | ppa004662 | 45 | 101 | 0.9813 | 8 | |
| P7 | ppa002552 | 93 | 117 | 0.976 | 1 | |
| P8 | ppa006076 | 90 | 102 | 0.95 | 7 | |
| P9 | ppa002787 | 211 | 96 | 0.8368 | 9 | |
| ^a^RPM or reads per million mapped reads derived from RNAseq libraries. | | | | | | |
| ^b^Efficiency derived from qPCR experiments with varying amounts of starting RNA. | | | | | | |
| ^c^R values from the efficiency experiments. | | | | | | |
| ^d^Overall ranking utilizing the RefFinder program. | | | | | | |
