# Supplementary material for: Identification of early fruit development reference genes in plum
Source: PLoS One. 2020 Apr 17;15(4):e0230920. doi: 10.1371/journal.pone.0230920 (PMC7164607; doi:10.1371/journal.pone.0230920)
Supplement: S6 Table — (DOCX) [file pone.0230920.s007.docx]

|  |  | | Table S6. Peach and Apple Orthologs. | | | |
| --- | --- | --- | --- | --- | --- | --- |
| Plum | Peach Orthologs | | Apple Orthologs | | | |
|  | V1.0 |  | GDV1.0 | | GDDH13V1.1 | |
| ID | Development | ID | Development | ID | Hormone Treatments | ID |
| P1 | ppa004809m | PeD1 | MDP0000912152 | AD1 | MD05G1102700 | AH1 |
|  |  |  | MDP0000881169 | AD2 | MD10G1108400 | AH2 |
| P2 | ppa009591m | PeD2 | MDP0000137420 | AD3 | MD13G1192800 | AH3 |
|  |  |  | MDP0000275687 | AD4 | MD16G1193500 | AH4 |
| P3 | ppa005747m | PeD3 | MDP0000127989 | AD5 | MD15G1358600 | AH5 |
|  |  |  | MDP0000242236 | AD6 | MD08G1173300 | AH6 |
| P4 | ppa017220m | PeD4 | MDP0000121874 | AD7 | MD11G1113000 | AH7 |
|  |  |  | MDP0000259167 | AD8 | MD03G1099100 | AH8 |
| P5 | ppa006628m | PeD5 | MDP0000173131 | AD9 | MD00G1004100 | AH9 |
|  |  |  | MDP0000765663 | AD10 | MD01G1193000 | AH10 |
| P6 | ppa004662m | PeD6 | MDP0000252152 | AD11 | MD16G1163000 | AH11 |
|  |  |  | MDP0000200406 | AD12 | MD13G1163500 | AH12 |
| P7 | ppa002552m | PeD7 | MDP0000120572 | AD13 | MD14G1029900 | AH13 |
|  |  |  | MDP0000232602 | AD14 | MD12G1029600 | AH14 |
| P8 | ppa006076m | PeD8 | MDP0000265765 | AD15 | MD12G1196100 | AH15 |
|  |  |  | MDP0000120387 | AD16 | MD04G1180800 | AH16 |
| P9 | ppa002787m | PeD9 | MDP0000813172 | AD17 | MD02G1148900 | AH17 |
|  |  |  | MDP0000251581 | AD18 | MD15G1263800 | AH18 |
