# Supplementary material for: Identification of early fruit development reference genes in plum
Source: PLoS One. 2020 Apr 17;15(4):e0230920. doi: 10.1371/journal.pone.0230920 (PMC7164607; doi:10.1371/journal.pone.0230920)
Supplement: S7 Table — (DOCX) [file pone.0230920.s008.docx]

| Table S7. Coefficient of Variance for RNAseq experiments in early Peach and Apple fruit for the nine candidate reference genes. | | | | | | | | | | | | | | | | | | | | | | | | | |
| --- | --- | --- | --- | --- | --- | --- | --- | --- | --- | --- | --- | --- | --- | --- | --- | --- | --- | --- | --- | --- | --- | --- | --- | --- | --- |
|  | P1 | | | P2 | | P3 | | | P4 | | | P5 | | P6 | | | P7 | | | P8 | | | P9 | |  |
| **Peach Development** | | | | | | | | | | | | | | | | | | | | | | | | | |
| Transcript | PeD1 | | | PeD2 | | PeD3 | | | PeD4 | | | PeD5 | | PeD6 | | | PeD7 | | | PeD8 | | | PeD9 | |  |
| All Libraries | 38 | | | 17 | | 23 | | | 20 | | | 19 | | 17 | | | 15 | | | 22 | | | 39 | |  |
| Hypanthium | 40 | | | 12 | | 10 | | | 13 | | | 14 | | 21 | | | 14 | | | 13 | | | 28 | |  |
| Ovary Wall | 10 | | | 6 | | 12 | | | 8 | | | 8 | | 16 | | | 5 | | | 10 | | | 11 | |  |
| Ovule | 13 | | | 11 | | 7 | | | 9 | | | 13 | | 12 | | | 8 | | | 14 | | | 10 | |  |
| Seed | 8 | | | 6 | | 6 | | | 13 | | | 7 | | 7 | | | 11 | | | 7 | | | 9 | |  |
| 0 day | 46 | | | 19 | | 28 | | | 21 | | | 12 | | 15 | | | 11 | | | 20 | | | 51 | |  |
| 5 day | 22 | | | 13 | | 22 | | | 23 | | | 24 | | 9 | | | 15 | | | 24 | | | 39 | |  |
| 12 day | 17 | | | 18 | | 24 | | | 20 | | | 21 | | 11 | | | 16 | | | 22 | | | 35 | |  |
| 20 day | 18 | | | 17 | | 20 | | | 18 | | | 16 | | 13 | | | 13 | | | 19 | | | 13 | |  |
| **Apple Development** | | | | | | | | | | | | | | | | | | | | | | | | | |
| Transcript | AD1 | AD2 | AD3 | AD4 | AD5 | | AD6 | AD7 | | AD8 | AD9 | AD10 | AD11 | | AD12 | AD13 | | AD14 | AD15 | | AD16 | AD17 | | AD18 |  |
| All Libraries | 24 | 24 | 18 | 15 | 27 | | 28 | 14 | | 19 | 152 | 45 | 34 | | 21 | 21 | | 13 | 28 | | 16 | 45 | | 61 |  |
| Hypanthium | 28 | 31 | 15 | 13 | 33 | | 15 | 15 | | 17 | 138 | 49 | 48 | | 24 | 15 | | 13 | 24 | | 18 | 36 | | 61 |  |
| Ovary Wall | 24 | 18 | 20 | 9 | 16 | | 10 | 12 | | 15 | 138 | 52 | 29 | | 22 | 7 | | 10 | 18 | | 12 | 48 | | 58 |  |
| Ovule | 8 | 14 | 14 | 12 | 10 | | 9 | 6 | | 11 | 6 | 20 | 19 | | 13 | 5 | | 5 | 13 | | 12 | 20 | | 16 |  |
| Seed | 10 | 16 | 7 | 10 | 7 | | 13 | 5 | | 4 | 18 | 7 | 6 | | 15 | 8 | | 5 | 3 | | 7 | 9 | | 22 |  |
| 0 day | 12 | 10 | 18 | 15 | 18 | | 29 | 15 | | 24 | 70 | 45 | 14 | | 16 | 14 | | 8 | 19 | | 20 | 54 | | 55 |  |
| 5 day | 12 | 17 | 14 | 14 | 28 | | 36 | 6 | | 21 | 20 | 26 | 20 | | 12 | 18 | | 9 | 20 | | 15 | 15 | | 28 |  |
| 12 day | 19 | 23 | 6 | 17 | 26 | | 29 | 11 | | 20 | 23 | 32 | 15 | | 18 | 27 | | 11 | 27 | | 11 | 12 | | 18 |  |
| 20 day | 33 | 39 | 14 | 8 | 33 | | 12 | 15 | | 7 | 29 | 31 | 28 | | 18 | 25 | | 19 | 35 | | 13 | 30 | | 34 |  |
| **Apple Hormone Treatments** | | | | | | | | | | | | | | | | | | | | | | | | | |
| Transcript | AH1 | AH2 | AH3 | AH4 | AH5 | | AH6 | AH7 | | AH8 | AH9 | AH10 | AH11 | | AH12 | AH13 | | AH14 | AH15 | | AH16 | AH17 | | AH18 |  |
| All Libraries | 31 | 25 | 45 | 29 | 38 | | 42 | 37 | | 30 | 44 | 61 | 51 | | 34 | 14 | | 16 | 31 | | 38 | 45 | | 42 |  |
| **Treatments by time** | | | | | | | | | | | | | | | | | | | | | | | | | |
| 18 DAA GA | 12 | 14 | 40 | 24 | 26 | | 30 | 24 | | 17 | 23 | 23 | 21 | | 14 | 13 | | 18 | 19 | | 13 | 10 | | 23 |  |
| 18 DAA HP | 39 | 10 | 20 | 21 | 24 | | 27 | 39 | | 13 | 28 | 35 | 11 | | 21 | 9 | | 12 | 50 | | 28 | 7 | | 9 |  |
| 18 DAA Neg | 32 | 13 | 17 | 32 | 22 | | 8 | 34 | | 31 | 24 | 24 | 13 | | 15 | 12 | | 12 | 23 | | 7 | 11 | | 4 |  |
| 18 DAA NAA | 6 | 8 | 11 | 14 | 13 | | 10 | 14 | | 11 | 8 | 14 | 20 | | 11 | 4 | | 4 | 7 | | 6 | 8 | | 10 |  |
| 132 DAA GA | 11 | 23 | 20 | 12 | 26 | | 29 | 18 | | 8 | 11 | 72 | 21 | | 17 | 9 | | 7 | 7 | | 10 | 18 | | 16 |  |
| 132 DAA HP | 30 | 51 | 47 | 29 | 29 | | 69 | 57 | | 60 | 49 | 72 | 61 | | 26 | 17 | | 22 | 14 | | 16 | 60 | | 58 |  |
| All GA | 16 | 19 | 49 | 29 | 32 | | 39 | 30 | | 14 | 40 | 57 | 45 | | 21 | 13 | | 16 | 17 | | 13 | 43 | | 43 |  |
| All HP | 34 | 34 | 45 | 25 | 27 | | 50 | 48 | | 38 | 52 | 74 | 51 | | 26 | 15 | | 18 | 37 | | 32 | 45 | | 38 |  |
| **Tissue by Treatment** | | | | | | | | | | | | | | | | | | | | | | | | | |
| GA Hypanthium | 25 | 19 | 28 | 19 | 27 | | 36 | 14 | | 11 | 56 | 87 | 55 | | 20 | 16 | | 9 | 26 | | 16 | 50 | | 37 |  |
| GA Ovary Wall | 12 | 8 | 38 | 26 | 9 | | 10 | 18 | | 13 | 33 | 43 | 37 | | 17 | 10 | | 15 | 11 | | 6 | 30 | | 30 |  |
| GA Ovule | 6 | 19 | 20 | 14 | 16 | | 19 | 14 | | 12 | 12 | 13 | 14 | | 8 | 6 | | 5 | 7 | | 11 | 6 | | 13 |  |
| HP Hypanthium | 43 | 23 | 33 | 20 | 20 | | 29 | 25 | | 16 | 53 | 88 | 46 | | 21 | 14 | | 8 | 28 | | 19 | 30 | | 19 |  |
| HP Ovary Wall | 9 | 9 | 42 | 22 | 16 | | 23 | 6 | | 10 | 48 | 54 | 34 | | 24 | 18 | | 7 | 17 | | 25 | 33 | | 24 |  |
| HP Ovule | 44 | 53 | 32 | 33 | 16 | | 21 | 62 | | 77 | 38 | 69 | 64 | | 27 | 13 | | 25 | 32 | | 29 | 65 | | 67 |  |
| NAA Hypanthium | 1 | 3 | 5 | 4 | 5 | | 4 | 8 | | 13 | 13 | 15 | 5 | | 11 | 3 | | 3 | 5 | | 3 | 5 | | 3 |  |
| NAA Ovary Wall | 7 | 11 | 11 | 22 | 13 | | 9 | 15 | | 10 | 4 | 5 | 13 | | 11 | 5 | | 5 | 6 | | 6 | 7 | | 4 |  |
| Neg Hypanthium | 50 | 19 | 20 | 48 | 24 | | 6 | 41 | | 39 | 35 | 28 | 17 | | 21 | 18 | | 17 | 36 | | 7 | 16 | | 3 |  |
| Neg Ovary Wall | 6 | 9 | 8 | 8 | 4 | | 1 | 9 | | 16 | 11 | 11 | 10 | | 4 | 2 | | 6 | 2 | | 1 | 4 | | 2 |  |
| **Tissue Only** | | | | | | | | | | | | | | | | | | | | | | | | | |
| Hypanthium | 38 | 23 | 38 | 30 | 53 | | 54 | 26 | | 22 | 52 | 73 | 58 | | 39 | 14 | | 12 | 25 | | 29 | 46 | | 38 |  |
| Ovary Wall | 20 | 11 | 41 | 27 | 31 | | 36 | 16 | | 17 | 40 | 59 | 41 | | 30 | 15 | | 14 | 17 | | 32 | 38 | | 34 |  |
| Ovule | 31 | 39 | 31 | 32 | 17 | | 20 | 63 | | 56 | 29 | 47 | 44 | | 28 | 13 | | 18 | 31 | | 35 | 44 | | 56 |  |
