# Supplementary material for: Identification of early fruit development reference genes in plum
Source: PLoS One. 2020 Apr 17;15(4):e0230920. doi: 10.1371/journal.pone.0230920 (PMC7164607; doi:10.1371/journal.pone.0230920)
Supplement: Attachment — Submitted filename: Response to Reviewers.docx [file pone.0230920.s009.docx]

Yun Zheng, Ph.D
Academic Editor
PLOS ONE

Dear Dr. Yun Zheng,

First, our thanks to the reviewers who pointed out several items that needed clarifying or improvement. We have made the suggested edits to the manuscript. In addition, we changed the naming as it seemed to get in the way of understanding what materials/genes we were working with. Below are the reviews and in bold, our response to the comments.

Response to Reviewers Comments:

Reviewer #1: In this manuscript, the authors tried to identify sets of genes that could be used to normalize expression studies in early fruit development, using RNAseq/qPCR experiments in a series of developmental plum fruit samples. They found nine newly identified candidate reference genes that appeared to be more consistent in the RNAseq data from early fruit libraries than the previously identified Prunus reference genes. Their results suggest that the re-mining of existing RNAseq data can find novel reference genes for a specific tissues or experimental conditions. I think this work is interesting and resourceful, the experiments and statistical analysis used meet the standard, and the manuscript is acceptable for publication.

Reviewer #2: This study used existing RNAseq data to identify standard genes to provide stable reference genes for early fruit development and endocarp tissue in plum. Nine candidate reference genes were chosen from a set of RNAseq data, three best genes as defined using RefFinder were used to normalize expression of a plum fruit developmental series and endocarp tissues from a normal endocarp plum and endocarp tissue from an endocarp mutant. Normalization with any of those three as well as a combination resulted in difference in endocarp expression in the mutant endocarp. It is an interesting study, however there still some concerns with the data.

1. The candidate reference gene P4 looks even stable between different library than P1 and P2 in figure 1, why don’t you choose it?

**Candidate Reference Gene P4 appears to have a compact distribution spread and good efficiency but was ranked 5^th^ in the Recommended Comprehensive ranking in RefFinder, with no 1^st^ place rankings in any of the programs. We focused on the top three genes in the Recommended Comprehensive ranking, which are all 1^st^ in at least one program. We added this to line 277 that we only proceeded with the top three genes and UBQ for the qPCR normalization experiments.**

2. Figure 1, the X axis legend don’t fit well with the Y axis data which make confusion.

**We have re-labeled the x-axis with the appropriate gene IDs. We have also changed the colors so that similar tissues are similar colors, and moved the legend so that this figure is more clear.**

3. Figure 3, the black line represents the non-normalized values, but the legend is P10. The Blue and grey line missed the legend. The X axis legend for P10 and p11 are partly missing. Please make sure the figures are presented scientifically and rigorously.

**We thank the reviewer for bringing this to our attention - the formatting of this figure was altered when it was uploaded. We have fixed the issues with the missing lines. We have changed the black line to “non-normalized” in the legend and we have simplified the legend to be shared between all four graphs. We also corrected S1 Fig which had similar issues.**

1. Is the manuscript technically sound, and do the data support the conclusions?
The manuscript must describe a technically sound piece of scientific research with data that supports the conclusions. Experiments must have been conducted rigorously, with appropriate controls, replication, and sample sizes. The conclusions must be drawn appropriately based on the data presented.
Reviewer #1: Yes
Reviewer #2: Yes

2. Has the statistical analysis been performed appropriately and rigorously?

Reviewer #1: Yes
Reviewer #2: No

**We hope that this has been clarified in the more correct presentation of the graphs. We reworded some of the results section to present a clearer picture of what we were doing. (lines 208-211; 217-227; 234-241) as well as retitling figure legend 3.**

3. Have the authors made all data underlying the findings in their manuscript fully available?

The [PLOS Data policy](https://gcc02.safelinks.protection.outlook.com/?url=http%3A%2F%2Fwww.plosone.org%2Fstatic%2Fpolicies.action%23sharing&data=02%7C01%7C%7C28a744198e3a47f4509e08d767caf2ef%7Ced5b36e701ee4ebc867ee03cfa0d4697%7C0%7C1%7C637091993664143330&sdata=6ioJfQHCBBvm4MH6W92Y9cGu%2F1y8Huw69GxtwPDOi5A%3D&reserved=0) requires authors to make all data underlying the findings described in their manuscript fully available without restriction, with rare exception (please refer to the Data Availability Statement in the manuscript PDF file). The data should be provided as part of the manuscript or its supporting information, or deposited to a public repository. For example, in addition to summary statistics, the data points behind means, medians and variance measures should be available. If there are restrictions on publicly sharing data—e.g. participant privacy or use of data from a third party—those must be specified.

Reviewer #1: Yes
Reviewer #2: Yes

**We are still in the process of submitting all the sequence data sets to SRA (NCBI). One set is completed but the other 4 are in the process. We will submit the accession numbers when we obtain all of them.**

4. Is the manuscript presented in an intelligible fashion and written in standard English?
PLOS ONE does not copyedit accepted manuscripts, so the language in submitted articles must be clear, correct, and unambiguous. Any typographical or grammatical errors should be corrected at revision, so please note any specific errors here.

Reviewer #1: Yes
Reviewer #2: Yes

**In addition to the suggested edits, we also made some cosmetic changes in order to improve the clarity of the manuscript. We thought this would help with the data interpretations.**

1. **We changed some of the abbreviations we assigned to genes.**

**Previously published reference genes are now referred to as REF1-REF13 instead of S1-S13. Candidate reference genes from plum are still referred to as P1-P9. The four plum genes that were used to test the normalization with P1-P9 are now referred to as “plum test” PT1-PT4 instead of P10-P15. Peach orthologs used to test normalization in the peach developmental series are now referred to as PeD1-PeD9. Apple orthologs from version 1 of the apple genome that are used to normalize the apple developmental series are now referred to as AD1-AD18 instead of D1-D18. Apple orthologs from the GDDH13 version of the apple genome used to normalize the apple hormone samples are now referred to as AH1-AH18 instead of H1-18.**

2. **We included frequent references to these abbreviations throughout the text in order to make it easier for the reader to keep track of them.**
3. **We changed the wording of some sentences/paragraphs to make them easier to understand.**

**Again, thank you for the helpful comments and we do hope that we have responded, they have improved the manuscript.**

Ann Callahan

RESEARCH GENETICIST

USDA-ARS,Appalachian Fruit Research Station

2217 Wiltshire Road,Kearneysville, WV 25430

Tel. (304)725-3451

FAX	(304)728-2340

Email ann.callahan@ars.usda.gov
